# Convex gradient optimization for increased spatiotemporal resolution and improved accuracy in phase contrast MRI

**DOI:** 10.1186/1532-429X-16-S1-W36

**Published:** 2014-01-16

**Authors:** Matthew J Middione, Holden H Wu, Daniel B Ennis

**Affiliations:** 1 Department of Radiological Sciences, University of California, Los Angeles, Los Angeles, California, USA; 2 Biomedical Physics Interdepartmental Program, University of California, Los Angeles, Los Angeles, California, USA

## Background

Decades of research have helped mitigate numerous sources of error in phase contrast MRI (PC-MRI), nevertheless chemical shift induced phase errors (CS errors) and spatiotemporal undersampling errors (STU errors) remain critical sources of error for which a cogent error mitigation strategy is needed. CS errors, which arise in PC-MRI because the complex signal for perivascular fat chemically shifts across the vessel wall and corrupts the complex blood signal, can be mitigated with an in-phase TE (TE_IN_) and a high receiver bandwidth [1]. STU errors arise from suboptimal spatiotemporal resolution. The objective was to design a PC-MRI sequence with improved sequence efficiency and evaluate the impact on mitigating both CS and STU errors.

## Methods

Hargreaves et al. [2] have shown that convex optimization (CVX) can be used to minimize gradient waveform durations subject to both hardware constraints (maximum available gradient amplitude and slew rate) and pulse sequence constraints (e.g. VENC, RF pulse, slice thickness, FOV, bandwidth, matrix size). We developed CVX PC-MRI to achieve improved spatiotemporal resolution to reduce STU errors while using the minimum TE_IN _(TE_IN,MIN_) to reduce CS errors for a fixed breath hold duration. Flow measurements were obtained at 3T (Siemens Trio) using a conventional flow compensated and flow encoded (FCFE) PC-MRI sequence and CVX PC-MRI optimized for high spatial resolution (CVX-SR) or high temporal resolution (CVX-TR). All sequences mitigated CS errors with a high receiver bandwidth and TEIN. CVX permits using TE_IN,MIN _= 2.46 ms while the FCFE sequence can only achieve TE_IN _= 4.92 ms. Total flow and peak velocity measurements were acquired in the ascending aorta (aAo), main pulmonary artery (PA), and right/left pulmonary arteries (RPA/LPA) of ten (N = 10) normal volunteers (Table 1).

**Table 1:** PC-MRI parameters.

	FCFE	CVX-SR	CVX-TR
TE_IN_/TR (ms)	4.92/7.00	2.46/4.00	2.46/3.95

Temporal resolution (ms)	55.9	56.1	31.6

VENC (cm/s)	150	150	150

Parallel acceleration	rate-2 GRAPPA with 24 reference lines		

Flip angle (degrees)	30	30	30

Segments	4	7	4

FOV (mm)	340 × 255	340 × 255	340 × 255

Pixel number	192 × 144	288 × 216	192 × 144

Pixel size (mm)	1.8 × 1.8	1.2 × 1.2	1.8 × 1.8

Bandwidth (Hz/px)	814	827	814

Duration (heart beats)	23	20	23

Acquired cardiac phases ^†^	13-18	13-18	23-32

^†^The number of acquired cardiac phases is heart rate dependent

## Results

The sequence efficiencies (readout duration/TR) were 17.7% for FCFE, 30.5% for CVX-SR, and 31.4% for CVX-TR. Measurements of total flow and peak velocity were significantly higher (P < 0.05) for CVX-SR and CVX-TR compared to FCFE (Table 2). On average, CVX-SR measured 8.1% higher total flow and 3.8% higher peak velocity and CVX-TR measured 5.1% higher total flow and 10.5% higher peak velocity.

**Table 2:** In vivo (N = 10) PC-MRI measures of total flow and peak velocity.

	FCFE	CVX-SR	CVX-TR
**Total Flow (mL) **			

aAo	89.0 ± 18.2	95.9 ± 19.4^†^	93.5 ± 17.9^†^

PA	92.6 ± 18.5	100.1 ± 20.1^†^	97.2 ± 18.0^†,‡^

RPA	48.1 ± 9.5	52.5 ± 9.9^†^	50.9 ± 9.5^†,‡^

LPA	44.3 ± 8.7	47.7 ± 10.2^†^	46.4 ± 9.0^†^

Peak Velocity (cm/s)			

aAo	117.2 ± 17.2	121.6 ± 15.3^†^	127.3 ± 15.8^†,‡^

PA	87.0 ± 11.8	90.0 ± 13.5^†^	95.4 ± 15.4^†,‡^

RPA	93.8 ± 15.9	97.1 ± 16.8^†^	104.7 ± 21.7^†,‡^

LPA	95.3 ± 18.0	99.7 ± 19.8^†^	107.2 ± 19.9^†,‡^

Data are expressed as mean ± standard deviation ^† ^P < 0.05 shows a statistical significant difference compared to FCFE. ^‡ ^P < 0.05 shows a statistical significant difference compared to CVX-SR.

## Conclusions

CVX PC-MRI nearly doubles sequence efficiency, reduces CS and STU errors, and produces more accurate measurements of blood flow and peak velocity. CVX-SR reports the highest total flow and CVX-TR reports the highest peak velocities, but further improvements in spatiotemporal resolution may still be needed for accurate quantification.

## Funding

This work was enabled by research support from Siemens Medical Solutions and the Department of Radiological Sciences to DBE.
